# Sex‐specific associations between cardiovascular risk factors and physical function: the Gambian Bone and Muscle Ageing Study

**DOI:** 10.1002/jcsm.13069

**Published:** 2022-11-08

**Authors:** Ayse Zengin, Mícheál Ó Breasail, Camille M. Parsons, Landing M. Jarjou, Ramatoulie E. Janha, Modou Jobe, Ann Prentice, Cyrus Cooper, Peter R. Ebeling, Kate A. Ward

**Affiliations:** ^1^ Department of Medicine, School of Clinical Sciences at Monash Health Monash University Clayton Victoria Australia; ^2^ MRC Nutrition and Bone Health Group Cambridge UK; ^3^ Ageing and Movement Research Group, Population Health Sciences, Bristol Medical School University of Bristol Bristol UK; ^4^ MRC Lifecourse Epidemiology Centre, Human Development and Health University of Southampton Southampton UK; ^5^ MRC Unit The Gambia London School of Hygiene and Tropical Medicine The Gambia; ^6^ National Institute for Health Research (NIHR) Musculoskeletal Biomedical Research Unit University of Oxford Oxford UK

**Keywords:** ageing, cardiovascular, fat, hand grip strength, peripheral vascular calcification, physical function, pulse pressure, The Gambia

## Abstract

**Background:**

In Sub‐Saharan Africa, the prevalence of obesity, cardiovascular disease (CVD) and impaired physical function are increasing due to rapid urbanization. We investigated sex differences in associations between cardiac workload, arterial stiffness, peripheral vascular calcification (PVC) and physical function in Gambian adults.

**Methods:**

A total of 488 Gambians aged 40–75+ years were recruited (men: 239; and women: 249). Supine blood pressure and heart rate were measured to calculate rate pressure product and pulse pressure. Presence of PVC was determined from tibia peripheral quantitative computed tomography scans. Physical function was assessed by chair rise test (CRT), single two‐legged jump (s2LJ) and hand grip strength (HGS). Body composition was measured by dual‐energy x‐ray absorptiometry; body size corrections were used to calculate fat mass index (FMI) and appendicular lean mass index (ALMI). Estimated glomerular filtration rate (eGFR) was measured from fasting blood samples. The relationship between rate pressure product, pulse pressure or presence of PVC (independent variable) with physical function parameters (dependent variable) was tested using linear regression. Sex‐interactions were tested (p‐int) adjusting for age, eGFR and ALMI/FMI. Results were expressed as mean differences between men and women with 95% confidence intervals. Mediation analyses used ALMI/FMI as mediator.

**Results:**

Women weighed less (54.7 kg ± 10.3 vs. 59.9 kg ± 10.3) and were shorter (157.8 cm ± 6.0 vs. 169.2 cm ± 7.0) compared with men (both *P* < 0.0001). Women had higher FMI (6.8 kg/m^2^ ± 2.9 vs. 2.9 kg/m^2^ ± 2.0, *P* < 0.0001) and eGFR (263.7 mL/min/1.73 m^2^ ± 133.1 vs. 237.6 mL/min/1.73 m^2^ ± 134.6), but lower ALMI (6.2 kg/m^2^ ± 0.7 vs. 8.02 kg/m^2^ ± 1.0, *P* < 0.0001) compared with men. There were significant mean differences between men and women in rate pressure product and s2LJ power (−1.08 [−1.21, −0.95]) and force (−0.57 [−0.63, −0.51]), only after adjusting for age, eGFR and FMI. There were significant mean differences in the associations between pulse pressure and CRT power (−0.28 [−0.31, −0.25]), s2LJ power (−1.07 [−1.20, −0.93]) and HGS (−11.94 [−13.35, −10.54]); these differences were greater after adjusting for age, eGFR and FMI, than ALMI. There were similar differences in the associations between PVC and physical function parameters. In men, FMI mediated the association between rate pressuree product and CRT power (*P* = 0.002), s2LJ force (*P* < 0.001) and s2LJ power (*P* = 0.001). ALMI did not mediate associations for either men or women.

**Conclusions:**

Multiple risk factors for CVD were associated with poorer physical function in men and were mediated by FMI. There is a need to identify strategies to slow/prevent the rising CVD burden and poor physical function in Sub‐Saharan Africa.

## Introduction

The prevalence of non‐communicable diseases (NCD) of ageing are rising globally and account for 70% of deaths annually; 80% occur in low‐ and middle‐income countries.^1^ The highest burden is among countries in Sub‐Saharan Africa,^2^, ^3^ mainly due to a rapid demographic transition characterized by increasing urbanization and changing lifestyle factors. With this transition, there is an increase in the prevalence of obesity, sarcopenia and cardiovascular disease (CVD)—consequently giving rise to multimorbidity. Declines in physical function have been associated with multimorbidity, with more severe consequences reported in low‐ to middle‐income countries, coupled to a higher economic healthcare burden.^4^

Variation in cardiovascular health potentially has a causal role in the heterogeneity of age‐related declines in physical function. The Whitehall II Study in adults aged 55–78 years residing in London, UK, reported that arterial stiffness was strongly associated with poorer physical function.^5^ More recently, data in adults aged 20–100 years from Berlin, Germany, show that for every 5‐kg decrease in handgrip strength, arterial stiffness (measured by pulse wave velocity) increased by 0.30 m/s (95% confidence interval [CI] [0.35, 0.25], *P* < 0.001).^6^ Data from the United States show that individuals who have peripheral vascular calcification (PVC) have impaired walking ability and insufficient physical activity levels.^7^, ^8^, ^9^ These findings contrast with the weak or absent links of blood pressure alone with physical function assessments. Comprehensive cardiac health assessments that provide insight into the different components of cardiac health (stiffness, PVC and myocardial oxygen consumption) may potentially provide a greater understanding of the associations between cardiovascular health and physical function in older adults.

Adiposity has been associated with an increased risk of NCDs, such as CVD^10^ and osteoporosis,^11^ and indirectly contribute to declines in physical function.^12^ Data from high‐income countries have shown that a high fat mass index (FMI) is associated with lower physical function compared with those with lower FMI.^13^ Furthermore, studies in Caucasian older adults from high‐income countries show that older women had higher FMI compared with men, which had greater implications on physical function.^14^, ^15^ There are no data from the African continent on the relationship between FMI and physical function and whether this differs between men and women.

Characterization of the association between cardiovascular health and physical function in the ageing population may help identify preventative strategies for CVD in Sub‐Saharan African countries such as the Gambia. Therefore, the aims of this study were to investigate the sex differences in (1) CVD risk factors and poor physical function (2) and whether this relationship is mediated via body composition.

## Study design

Participants were from the Gambian Bone and Muscle Ageing Study (GamBAS), a longitudinal prospective observational study investigating musculoskeletal ageing in men and women from a rural region of the Gambia^16^ (ISRCTN17900679). The Kiang West Demographic Surveillance System^17^ was initially used to identify men and women >40 years residing within the four core survey villages of this region. After initial village sensitization and discussion on the design of the study with the village elders, participants were located and approached by local fieldworkers who explained the study in the local language and invited them to participate.^16^ Stratified sampling was used for recruitment to ensure equal distribution of participants across each sex and 5‐year age bands, namely, 40–44.99, 45–49.99, 50–54.99, 55–59.99, 60–64.99, 65–69.99, 70–74.99, and 75 years and over. To reach target group sizes, individuals from six other villages in the area were also recruited; each age band had 25–35 men and women. The sample size calculation was based on the primary outcome of within individual change in femoral neck bone mineral density over a 1.5–2‐year follow‐up.^16^ The population of Kiang West is mainly of Mandinka ethnicity (80%) living across approximately 36 villages.^17^ Villages are divided into compounds, where extended multi‐generational families live together. The main livelihood is rural subsistence farming; income and food availability/access fluctuate according to the annual rainy season (June to October).^17^ This study was conducted according to the guidelines laid down in the Declaration of Helsinki, and all procedures involving research study participants were approved by the joint Gambia Government/MRC The Gambia Ethics Committee (#SCC1222).

Written informed consent was obtained from all participants. Frail, pregnant or lactating individuals were excluded. All outcomes were measured at a single research visit; the current study is a cross‐sectional analysis of secondary outcomes. Weight (kg) was measured using electronic scales and standing height (cm) was measured using a stadiometer.

### Blood pressure

Participants' blood pressure was measured using the OMRON 705IT blood pressure monitor (OMRON Healthcare Europe B.V. Hoofddorp, The Netherlands). Participants were in a supine position and rested for 5 min, and then blood pressure measured in triplicate; the mean of the three measurements were used for analysis. Systolic blood pressure (SBP, mmHg), diastolic blood pressure (DBP, mmHg) and heart rate (beats per minute, bpm) were recorded. Rate pressure product was then calculated as heart rate multiplied by SBP (bpm × mmHg).^18^ Rate pressure product is an indication of cardiac workload, where a higher rate pressure product corresponds to a higher cardiac workload.^19^ Pulse pressure, a measure of arterial stiffness, was calculated as the difference between SBP and DBP.^20^ Hypertension was defined as SBP ≥ 140 mmHg and/or a DBP ≥ 90 mmHg.^21^

### Dual‐energy x‐ray absorptiometry

Each participant had a whole‐body scan by dual‐energy x‐ray absorptiometry (DXA) as previously described,^22^ (GE Lunar Prodigy, software version 10, GE Medical, Waltham, MA, USA). Body composition outcomes were as follows: total fat mass (kg), total lean mass (kg) and appendicular lean mass (ALM), which was calculated as arms plus legs lean mass (kg). Usually, taller individuals have greater lean mass; therefore, we assessed lean mass as ALM index (ALMI), which was calculated as ALM divided by height squared (m^2^)^23^, ^24^; similarly, FMI was calculated as whole body fat mass divided by height squared (m^2^). Scans were excluded due to motion artefact, a participant with leprosy and one with geophagy.

### Peripheral quantitative computed tomography

Peripheral quantitative computed tomography (pQCT) measurements were made and scans analysed, as previously described, at the tibia using a Stratec XCT‐2000 scanner (Stratec, Pforzheim, Germany), software version 6.20c.^25^ All measurements were taken in the non‐dominant limb. Leg length was measured with a tape measure and defined as the distance from the most proximal edge of the medial malleolus to the intercondylar eminence. Measurements were taken at the 4%, 38% and 66% of the leg length. As an indicator of peripheral artery disease, the presence of PVC was quantified by visualizing calcium plaque(s) as a circular area of high attenuation equivalent to bone in the pQCT images. Participants who had at least one visualized calcium plaque found in any of the scans taken at the tibia were considered to have calcified vessels, while those who had none served as having no calcified vessels. Scans were excluded where significant motion artefact was detected and distorted the image. We modified a previously published criterion to determine the presence of PVC in tibia scans from HRpQCT^26^, ^27^ that is, linear or tubular hyper‐dense zones of circular, semicircular, or crescent‐like structures that corresponded to anatomical territories of the anterior tibial artery, posterior tibial artery, interosseous branches, or smaller intramuscular or subcutaneous arterioles.

### Jumping mechanography

To assess physical function, a Leonardo Mechanography Ground Reaction Force Platform (Leonardo software version 4.2; Novotec Medical GmbH, Germany) was used.^28^ Participants were asked to perform two tests: a single two‐legged countermovement jump (s2LJ) and a chair rise test (CRT).^29^ Participants performed each test three times. Briefly, for the s2LJ, individuals were asked bend their knees to jump as high as possible and the jump with highest height used for analysis. Force (kN) and power (kW) were measured. For the CRT, individuals were asked to stand from a chair that was anchored to the platform without using their arms. Individuals who were able to complete the single chair stand were asked to repeat the chair rise test three times as quickly as possible, with the quickest CRT taken for analysis. The time taken to complete the CRT (s) and the power during the CRT (kW) were recorded. The intra‐ and inter‐rater reliability of jumping mechanography has been reported^30^, ^31^ with a CV of 0.3%–0.6% in 10 healthy adults.

### Hand grip strength

Hand grip strength was measured using a hand dynamometer (Jamar Hand Dynamometer, Patterson Medical, Bolingbrook, IL, USA).^32^ The individual was seated in an upright position with the arm supported on the armrest of the chair with the wrist in a neutral position and the thumb facing upwards. Participants were instructed to exert maximal force. For each individual, the first of four measurements was regarded as a practice, and the maximum force (kg) in the following three measurements was recorded as the participant's hand grip strength.

### Blood collection

Fasting venous blood samples were collected in lithium heparin tubes in the morning from each participant, and samples were stored at −80°C. Plasma was separated by centrifugation at ×1800 *g* for 10 min at 4°C, and subsequently transported for analysis to the MRC Elsie Widdowson Laboratory, Cambridge, UK, on dry ice and stored at −80°C. Creatinine was measured using the enzymatic creatinine assay on the Konelab analyser (Thermo Fischer Scientific, Finland). Estimated glomerular filtration rate (eGFR, mL/min/1.73 m^2^) was calculated using the 2021 CKD‐EPI Creatinine Equation.^33^

### Data analysis

All analyses were performed in Stata, Version 15.0 (StataCorp, College Station, TX, USA); statistical significance was set at *P* < 0.05. Descriptive statistics were used to describe participant characteristics and are presented as mean ± standard deviation (SD), and categorical variables as frequency (*n*) and percentage (%). Between‐group differences in the participant characteristics were tested with a one‐way ANOVA or *χ* ^2^ test for categorical variables.

We explored the relationship between rate pressure product, pulse pressure or presence of PVC (independent variable) with physical function parameters (dependent variable) using linear regression with adjustments for *a priori* confounders age, eGFR and ALMI; and age, eGFR and FMI. To test if these relationships were different between sex, we included a sex × outcome (either rate pressure product or pulse pressure or presence of PVC) interaction term and reported the mean differences and the relevant *P* value (p‐int). For these regression analyses described, rate pressure product or pulse pressure was transformed to natural logarithms to normalize distributions; the results were expressed as beta coefficients with 95% confidence intervals.^34^ For the regressions with the sex × PVC interaction term, the mean differences between those without compared with those with PVC were expressed within sex using a post‐hoc command to test within group differences (e.g., men without PVC vs. men with PVC; women without PVC vs. women with PVC).

Mediation analyses were used to examine whether associations between rate pressure product and physical function parameters, were mediated via FMI or ALMI. These analyses were repeated to determine the associations between pulse pressure/PVC and physical function parameters. Mediation analyses were carried out separately in men and women. To generate robust 95% confidence intervals for path analyses, bootstrapping was performed by randomly sampling the data with replacement with a sample size equal to the original, thereby generating 5000 replications. We report the indirect effect size with the corresponding *P* value.

## Results

In total, 488 participants were recruited (239 men). On average, women weighed less and were shorter compared with men (Table 1). Women had higher total fat content and FMI (both *P* < 0.0001), while men had higher total lean mass, ALM and ALMI (all *P* < 0.0001; Table 1). Mean eGFR levels were higher in women than in men. Mean rate pressure product and heart rate were higher in women compared with men (*P* < 0.001), with no sex differences in mean SBP, DBP, pulse pressure or hypertension prevalence. There were no sex differences in CRT time, but CRT power, s2LJ force and power, and hand grip strength were all higher in men than in women (all *P* < 0.0001; Table 1).

**Table 1:** Participant characteristics

	Men (*n* = 239)	Women (*n* = 249)	*P* value
General characteristics			
Age (year)	60.8 ± 12.3	61.1 ± 12.5	0.798
Weight (kg)	59.9 ± 10.3	54.7 ± 10.3	<0.0001
Height (cm)	169.2 ± 7.0	157.8 ± 6.0	<0.0001
BMI (kg/m^2^)	20.9 ± 3.1	21.9 ± 3.7	0.0009
Waist‐to‐height ratio	0.460 ± 0.058	0.455 ± 0.051	0.086
Total fat mass (kg)	8.3 ± 6.0^(*n* = 235)^	17.1 ± 7.4^(*n* = 242)^	<0.0001
Total percent fat (%)	13.0 ± 7.3^(*n* = 235)^	30.0 ± 8.0^(*n* = 242)^	<0.0001
FMI (kg/m^2^)	2.9 ± 2.0^(*n* = 248)^	6.8 ± 2.9^(*n* = 242)^	<0.0001
Total lean mass (kg)	49.0 ± 6.2^(*n* = 235)^	35.0 ± 4.3^(*n* = 242)^	<0.0001
ALM (kg)	22.9 ± 3.6^(*n* = 238)^	15.6 ± 2.4^(*n* = 248)^	<0.0001
ALMI (kg/m^2^)	8.0 ± 1.0^(*n* = 238)^	6.2 ± 0.7^(*n* = 248)^	<0.0001
eGFR (mL/min/1.73 m^2^)	237.6 ± 134.6	263.7 ± 133.1	0.032
Cardiac parameters			
SBP (mmHg)	132.8 ± 23.1	135.1 ± 22.7	0.268
DBP (mmHg)	72.2 ± 10.5	73.5 ± 10.3	0.192
Heart rate (bpm)	64.3 ± 12.4	68.7 ± 11.6^(*n* = 244)^	0.0001
Rate pressure product (bpm × mmHg)	8500 ± 2094	9274 ± 2228^(*n* = 241)^	0.0004
Pulse pressure (mmHg)	60.5 ± 17.4	61.6 ± 17.2	0.496
MAP (mmHg)	92.4 ± 13.6	94.0 ± 13.3	0.192
Hypertension prevalence *n* (%)	71 (29.7)	91 (36.6)	0.109
Calcified vessels *n* (%)	59 (26.6) ^(*n* = 222)^	52 (22.5) ^(*n* = 231)^	0.601
Physical function parameters			
CRT time (s)	5.1 ± 1.7 ^(*n* = 237)^	5.2 ± 1.8 ^(*n* = 246)^	0.632
CRT power (kW)	0.54 ± 0.2 ^(*n* = 236)^	0.34 ± 0.1 ^(*n* = 246)^	<0.0001
s2LJ force (kN)	1.5 ± 0.4 ^(*n* = 208)^	1.2 ± 0.3 ^(*n* = 189)^	<0.0001
s2LJ power (kW)	1.8 ± 0.9 ^(*n* = 208)^	1.0 ± 0.4 ^(*n* = 189)^	<0.0001
Hand grip strength (kg)	31.3 ± 9.2	20.3 ± 5.8	<0.0001

*Note:* Values are mean ± standard deviation. Hypertension classified as SBP > 140 mmHg or DBP > 80 mmHg.

Abbreviations: ALM, appendicular lean mass; ALMI, appendicular lean mass index; BMI, body mass index; CRT, chair rise test; DBP, diastolic blood pressure; eGFR, estimated glomerular filtration rate; FMI, fat mass index; MAP, mean arterial pressure; s2LJ, single two‐legged jump; SBP, systolic blood pressure.

**Figure 1:**
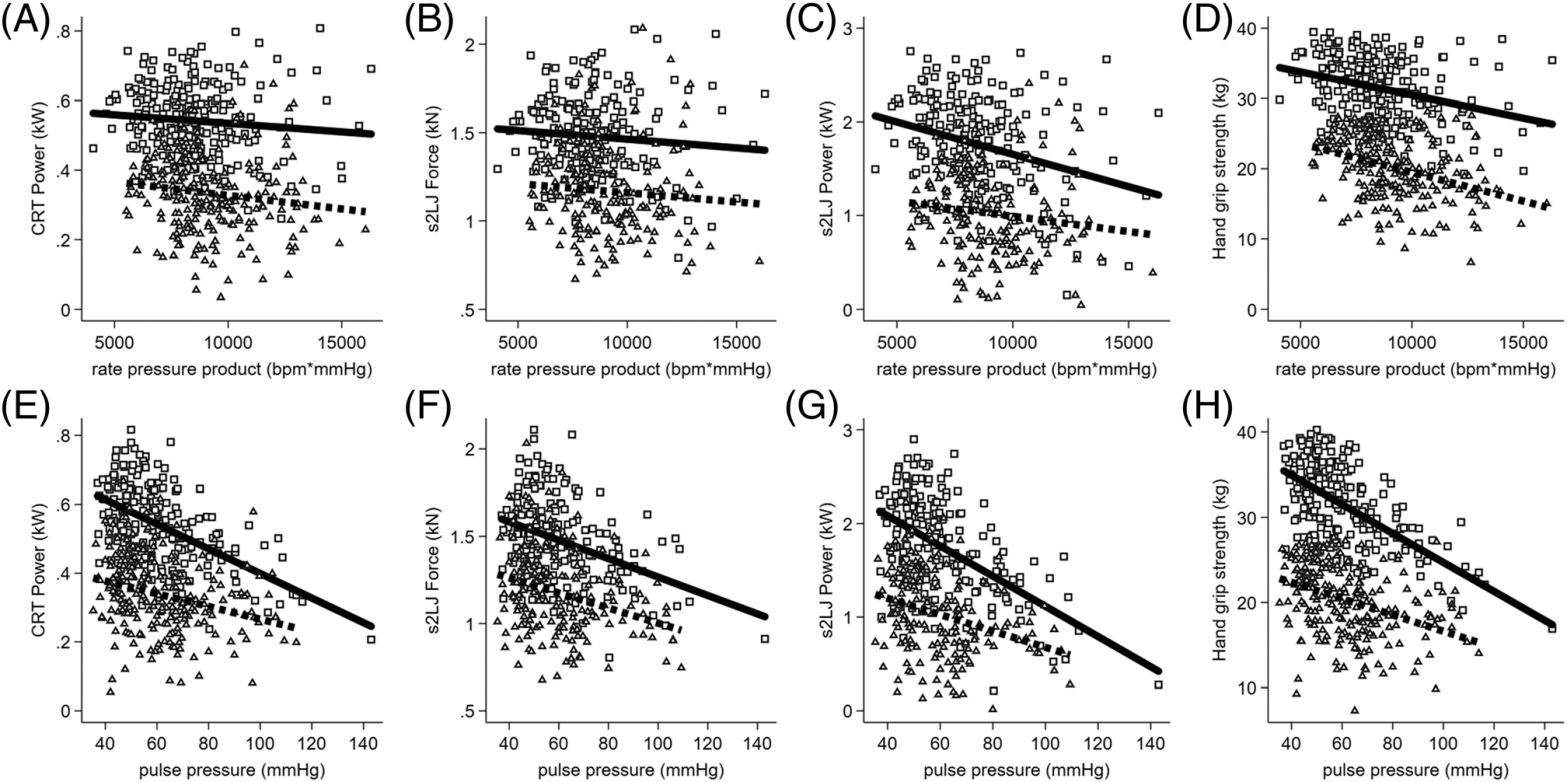
Relationship between rate pressure product (A–D) and pulse pressure (E–H) with physical function parameters in Gambian men and women. Scatter plots included a sex × rate pressure product or pulse pressure interaction for which the *P* values were reported (p‐int) and were adjusted for age and fat mass index. Squares and solid line represent men, triangles and dashed line represent women. Chair rise test (CRT), single two‐legged jump (s2LJ).

In unadjusted analyses, there were no sex differences in the associations between rate pressure product and physical function parameters (Table 2). Following adjustments for age, eGFR and ALMI, there were sex differences in the associations between rate pressure product and s2LJ power (pint = 0.003) (Table 2). In addition to this, following adjustments for age, eGFR and FMI, there were also sex differences in the association between in rate pressure product and s2LJ force where there was a greater negative difference in men but not in women (Table 2, Figure 1A–D). There were sex differences in the association between pulse pressure and hand grip strength with a greater negative difference in men than in women (−10.82 [−12.12, −9.53]); adjusting for age, eGFR and ALMI further revealed differences in CRT power and s2LJ power (Table 2). These sex differences remained after adjusting for age, eGFR and FMI and revealed additional differences to a greater magnitude in s2LJ force (Table 2, Figure 1E–H).

**Table 2:** The relationship between physical function with cardiac parameters (rate pressure product, pulse pressure and presence of calcified vessels)

		Unadjusted		Adjusted for age, eGFR + ALMI		Adjusted for age, eGFR + FMI	
		Mean difference	*p‐int*	Mean difference	*p‐int*	Mean difference	*p‐int*
Rate pressure product	CRT time (s)	−0.02 (−0.34, 0.30)	0.724	−0.12 (−0.55, 0.30)	0.708	−0.01 (−0.38, 0.36)	0.610
	CRT power (kW)	−0.20 (−0.24, −0.17)	0.742	−0.06 (−0.10, −0.03)	0.232	−0.29 (−0.32, −0.26)	0.121
	s2LJ force (kN)	−0.30 (−0.37, −0.24)	0.938	0.05 (−0.01, 0.12)	0.142	−0.57 (−0.63, −0.51)	0.044*
	s2LJ power (kW)	−0.71 (−0.85, −0.56)	0.324	−0.17 (−0.31, −0.02)	0.003*	−1.08 (−1.21, −0.95)	0.004*
	Hand grip strength (kg)	−10.61 (−11.98, −9.23)	0.748	−7.03 (−8.62, −5.44)	0.292	−11.93 (−13.37, −10.49)	0.524
Pulse pressure	CRT time (s)	0.06 (−0.25, 0.37)	0.143	−0.08 (−0.50, 0.34)	0.405	−0.03 (−0.40, 0.34)	0.529
	CRT power (kW)	−0.20 (−0.23, −0.17)	0.065	−0.05 (−0.09, −0.02)	<0.0001*	−0.28 (−0.31, −0.25)	<0.0001*
	s2LJ force (kN)	−0.30 (−0.37, −0.24)	0.632	0.06 (−0.002, 0.12)	0.199	−0.56 (−0.62, −0.50)	0.039*
	s2LJ power (kW)	−0.73 (−0.87, −0.60)	0.072	−0.16 (−0.30, −0.02)	0.002*	−1.07 (−1.20, −0.93)	0.001*
	Hand grip strength (kg)	−10.82 (−12.12, −9.53)	0.034*	−7.02 (−8.55, −5.59)	<0.0001*	−11.94 (−13.35, −10.54)	0.001*
Presence of calcified vessels	CRT time (s)	0.17 (−0.15, 0.49)	0.919	−0.04 (−0.47, 0.40)	0.679	0.08 (−0.31, 0.47)	0.671
	CRT power (kW)	−0.21 (−0.25, −0.18)	0.292	−0.06 (−0.10, −0.02)	0.159	−0.29 (−0.33, −0.26)	0.017*
	s2LJ force (kN)	−0.32 (−0.38, −0.25)	0.360	0.05 (−0.02, 0.12)	0.503	−0.57 (−0.63, −0.51)	0.028*
	s2LJ power (kW)	−0.77 (−0.91, −0.63)	0.071	−0.18 (−0.33, −0.03)	0.035*	−1.09 (−1.23, −0.95)	0.004*
	Hand grip strength (kg)	−11.44 (−12.80, −10.09)	0.082	−7.23 (−8.85, −5.62)	0.018*	−12.33 (−13.83, −10.84)	0.015*

*Note:* Data are expressed as mean differences between men and women with 95% confidence intervals. The p‐int values are from sex × physical function interactions.

Abbreviations: CRT, chair rise test; ALMI, appendicular lean mass index; eGFR, estimated glomerular filtration rate; FMI, fat mass index; s2LJ, single two‐legged jump.

* *P* < 0.05.

The presence of PVC in men was negatively associated with s2LJ power (*P* = 0.035) and hand grip strength (*P* = 0.018) only after adjustments for age, eGFR and ALMI (Table 2). In addition to these associations, following adjustments for age, eGFR and FMI, there were greater negative differences in men with CRT power (−0.29 [−0.33, −0.26]) and s2LJ force (−0.57 [−0.63, −0.51]) when compared with women (Table 2).

**Figure 2:**
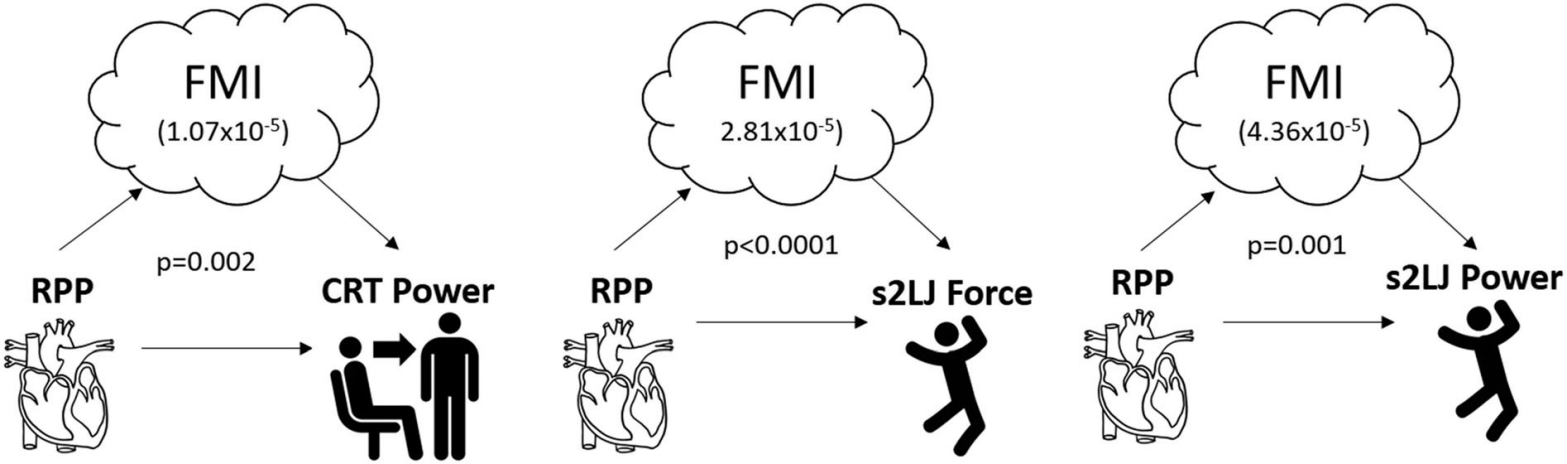
Mediation analyses of rate pressure product (independent variable) on physical function parameters (dependent variable) via FMI (mediator) in men. Indirect effect sizes and corresponding *P* values are reported. RPP, rate pressure product; CRT, chair rise test; s2LJ, single two‐legged jump; FMI, fat mass index.

In men, mediation analysis revealed the negative associations between rate pressure product and physical function parameters were mediated through FMI (Figure 2). The mediation effect of FMI on the association between rate pressure product: CRT power (1.07 × 10^−5^, *P* = 0.002), s2LJ force (2.81 × 10^−5^, *P* < 0.0001) and s2LJ power (4.36 × 10^−5^, *P* = 0.001). There was no mediation effect of ALMI on the association between rate pressure product and physical function parameters. There was no mediation of FMI or ALMI on the associations between pulse pressure/PVC and physical function. In women, there was no mediation of ALMI or FMI on any of the associations between cardiovascular health and physical function.

## Discussion

In this study we report sex differences in the associations between rate pressure product, pulse pressure (indicators of cardiac workload and arterial stiffness, respectively), and PVC with physical function parameters in a rural population of Gambian men and women aged 40–75+ years. Rate pressure product, pulse pressure and PVC were all negatively associated with physical function in men, with no differences in women. In men, these effects were mediated via FMI and not ALMI, while there was no mediation by either FMI or ALMI in women.

Higher mean heart rate in women compared with men may indicate increased sympathetic activity.^35^ Data shows higher levels of sympathetic activity with age in women compared with men.^36^ In line with this, high sympathetic activity has been associated with better physical function.^13^, ^14^ These findings suggest that higher sympathetic activity may be protective in Gambian women and may lessen the magnitude of the age‐associated declines in physical function.

Our data show the negative associations of rate pressure product, pulse pressure and PVC on physical function were observed only in Gambian men. This may partly be explained by the smaller amount of variability in this population of men especially body composition, compared with women. Indeed, we have previously reported that with increasing age there is a greater magnitude of variation in body composition (fat and lean mass) in women than in men.^16^ Gambian men exhibited similar BMI among the cohort, which is in contrast to the women who displayed a wider distribution in BMI. Factors contributing to the higher variation in lifestyle or life events in Gambian women may include parity, sex hormones and other factors such as diet, physical activity and living arrangements.

Gambian men with PVC had greater negative associations with physical function parameters. Plaque build‐up in the arteries occurs over time and is likely coupled to other changes within the body (increased fat mass, fat infiltration of muscle), which may contribute to increases in sedentary time—a contributor to physical function decline. Triaxial accelerometers are established objective tools in assessing physical activity. We previously reported that rate pressure product and pulse pressure were worse in women with PVC compared with those without PVC, while there were no differences in these parameters in Gambian men with and without PVC.^27^ Longitudinal data from GamBAS will help determine whether men who had PVC at baseline demonstrate even further deterioration in physical function at follow‐up; ankle–brachial index will also be measured—an independent predictor of incident CVD events and CVD,^37^ and subsequently will allow for a further comprehensive investigation of interactions between PVC, ankle–brachial index and physical function in Gambian adults.

FMI mediated the negative association between rate pressure product and physical function in Gambian men and not in women. The magnitude of this mediation was modest, and it is possible that we did not have the required statistical power to detect a robust mediation. Additionally, our cross‐sectional analysis does not allow us to infer causation; nevertheless, we hypothesize that increases in rate pressure product in turn increase FMI, which then leads to poorer physical function. The population are being followed‐up, which will potentially allow us to determine whether differences in cardiovascular risk factors cause declines in physical function via changes in FMI. There may be sex differences in systemic factors that affect FMI. For instance, low levels of high‐density lipoprotein (HDL) are strongly associated with obesity (higher BMI and waist‐to‐hip ratio) in European populations contributing to higher risk of CVD; in contrast, populations from Sub‐Saharan Africa have low HDL levels, even in those who are thin rural dwellers and with low BMI.^38^ Furthermore, these ethnic differences appear to be multifaceted as data from rural populations in Uganda and Malawi show sex differences in the prevalence of low HDL (women: 69%–77% vs. men: 41%–59%), while the opposite has been reported in populations from the United Kingdom.^38^ Low adiponectin levels have been shown to be an independent risk factor for CVD,^39^ and although the relationship between age and adiponectin is complex,^40^ adiponectin may affect the mediation of FMI. Additionally, adiponectin levels have been positively associated with total cholesterol and HDL and negatively associated with low‐density lipoprotein (LDL) and triglycerides, indicating a role in dyslipidaemia rather than obesity alone.^41^ Lipid panel data and adiponectin levels are not available in this cohort yet warrant further investigation.

The suite of measurements in our study are unique to Sub‐Saharan Africa and allow detailed characterization of musculoskeletal health. Our set of physical function parameters include not only handgrip strength but also CRT and s2LJ that require coordinated action of several different physical systems in addition to musculoskeletal, such as the nervous, vestibular and cardiopulmonary systems. There are potential limitations in this study. The observational design does not allow us to draw conclusions about causality or within individual change; the cohort is being followed‐up, which will potentially allow us to determine whether differences in cardiovascular risk factors cause declines in physical function. However, the sources of these associations are multifaceted, resulting from the complex interplay between age‐related biological changes, life‐course social and economic conditions and environmental exposures—data that we do not have. The primary outcome of GamBAS was to determine the changes in bone mineral density in men and women; the current study is a cross‐sectional analysis of secondary outcomes. Kiang West is one region of the Gambia, and the data may not be generalizable to urban areas of the country, to other West African countries or to the remainder of the continent. We did not collect objective data on physical activity (accelerometers) and so we do not know whether this may have been a factor in the sex differences reported. As an indirect measure of ‘manual’ physical activity, data from questionnaires revealed that a similar proportion of men and women were involved in farm or field work, that is, 80% and 85%, respectively.^16^ Also, hormonal changes in women due to menopause may have influenced the relationships between risk factors of cardiovascular health and physical function. We were unable to ascertain accurately menopausal status in our study population and acknowledge this would be important to determine in future studies. Nutrition is another factor that is important for physical function, which may have contributed to the sex differences. In previous work, we report that overall, men had higher intakes of all micronutrients; notable sex differences include 21% and 33% greater daily habitual calcium and iron intake in men compared with women, respectively.^16^ Additionally, data from the Gambian population have shown that 25OHD concentrations, a key nutrient involved in musculoskeletal and cardiovascular health, were sufficient.^42^, ^43^ Medication use is limited in this cohort, due to a lack of affordability and accessibility across the Gambia. Treatment for hypertension is rarely in the form of beta‐blockers and estimated to be less than 5%; whereas the most commonly prescribed are calcium channel blockers, thiazide diuretics and angiotensin‐converting enzyme inhibitors. Despite low use, treatment of CVD by these medications may have confounded our findings. We could not adjust for potential confounders such as diabetes mellitus, as there is a lack of detailed and complete data in this rural study setting in West Africa. However, as a marker of renal function, we adjusted our analyses for eGFR.

In conclusion, there are sex differences in the associations between cardiovascular risk factors and physical function in a rural population of Gambian men and women aged over 40 years. Multiple risk factors for CVD are associated with poorer physical function in rural Gambian men and not in women.

## Conflicts of interest

Ayse Zengin declares that she has no conflict of interest. Mícheál Ó Breasail declares that he has no conflict of interest. Camille M. Parsons declares that she has no conflict of interest. Landing M. Jarjou declares that he has no conflict of interest. Ramatoulie E. Janha declares that she has no conflict of interest. Modou Jobe declares that he has no conflict of interest. Ann Prentice declares that she has no conflict of interest. Cyrus Cooper declares that he has no conflict of interest. Peter R Ebeling declares that he has no conflict of interest. Kate A. Ward declares that she has no conflict of interest.
